# Zika virus transmission by Brazilian *Aedes aegypti* and *Aedes albopictus* is virus dose and temperature-dependent

**DOI:** 10.1371/journal.pntd.0008527

**Published:** 2020-09-08

**Authors:** Thais Chouin-Carneiro, Mariana Rocha David, Fernanda de Bruycker Nogueira, Flavia Barreto dos Santos, Ricardo Lourenço-de-Oliveira

**Affiliations:** 1 Laboratório de Mosquitos Transmissores de Hematozoários, Instituto Oswaldo Cruz, Fiocruz, Rio de Janeiro, RJ, Brazil; 2 Laboratório de Imunologia Viral, Instituto Oswaldo Cruz, Fiocruz, Rio de Janeiro, RJ, Brazil; Fundacao Oswaldo Cruz, BRAZIL

## Abstract

**Background:**

Zika virus (ZIKV) emerged in the Pacific Ocean and subsequently caused a dramatic Pan‐American epidemic after its first appearance in the Northeast region of Brazil in 2015. The virus is transmitted by *Aedes* mosquitoes. We evaluated the role of temperature and infectious doses of ZIKV in vector competence of Brazilian populations of *Ae*. *aegypti* and *Ae*. *albopictus*.

**Methodology/Principal findings:**

Two *Ae*. *aegypti* (Rio de Janeiro and Natal) and two *Ae*. *albopictus* (Rio de Janeiro and Manaus) populations were orally challenged with five viral doses (10^2^ to 10^6^ PFU / ml) of a ZIKV strain (Asian genotype) isolated in Northeastern Brazil, and incubated for 14 and 21 days in temperatures mimicking the spring-summer (28°C) and winter-autumn (22°C) mean values in Brazil. Detection of viral particles in the body, head and saliva samples was done by plaque assays in cell culture for determining the infection, dissemination and transmission rates, respectively. Compared with 28°C, at 22°C, transmission rates were significantly lower for both *Ae*. *aegypti* populations, and *Ae*. *albopictus* were not able to transmit the virus. *Ae*. *albopictus* showed low transmission rates even when challenged with the highest viral dose, while both *Ae*. *aegypti* populations presented higher of infection, dissemination and transmission rates than *Ae*. *albopictus*. *Ae*. *aegypti* showed higher transmission efficiency when taking virus doses of 10^5^ and 10^6^ PFU/mL following incubation at 28°C; both *Ae*. *aegypti* and *Ae*. *albopictus* were unable to transmit ZIKV with virus doses of 10^2^ and 10^3^ PFU/mL, regardless the incubation temperature.

**Conclusions/Significance:**

The ingested viral dose and incubation temperature were significant predictors of the proportion of mosquito’s biting becoming infectious. *Ae*. *aegypti* and *Ae*. *albopictus* have the ability to transmit ZIKV when incubated at 28°C. However Brazilian populations of *Ae*. *aegypti* exhibit a much higher transmission potential for ZIKV than *Ae*. *albopictus* regardless the combination of infection dose and incubation temperature.

## Author summary

Zika virus is an arbovirus that has become endemic in Brazilian territory and in tropical and subtropical countries of the Americas since 2015. The virus is transmitted by *Aedes* mosquitoes. *Aedes aegypti* and *Aedes albopictus* are widespread in Brazil. To evaluated the influence of temperature and the effect of the infectious dose of ZIKV in vector competence, Brazilian populations of *Ae*. *aegypti* and *Ae*. *albopictus* were orally exposed to different infectious doses, distributed from 10^2^ to 10^6^ PFU / ml and incubated at 22°C and 28°C. We experimentally demonstrated that both populations of *Ae*. *aegypti* and *Ae*. *albopictus* have the ability to transmit ZIKV when incubated at 28°C, however the infectious dose strongly influenced the proportion of mosquitoes that were able to transmit the virus. *Ae*. *albopictus* populations showed low transmission rates when challenged with the highest viral dose, while *Ae*. *aegypti* populations are more susceptible, presenting high rates of infection, dissemination and transmission. When incubated at 22°C, *Ae*. *albopictus* populations were not able to transmit the virus. Combined, the results indicate that Brazilian populations of *Ae*. *aegypti* exhibit a much higher transmission potential for ZIKV than *Ae*. *albopictus*.

## Introduction

Zika virus (ZIKV) has recently emerged as a global public health emergency of international concern. ZIKV belongs to the *Flavivirus* genus, which also includes other important human pathogens such as dengue fever (DENV), yellow fever (YFV), West Nile (WNV), Japanese encephalitis (JEV), and tick borne encephalitis viruses (TBEV) [1]. The viral genome of ZIKV consists of an enveloped non-segmented, single-stranded, positive-sense RNA, which encodes three structural proteins (C, PrM, and E) and seven non-structural proteins (NS1, NS2A, NS2B, NS3, NS4A, NS4B, NS5) [2].

ZIKV was first identified in the Zika forest in Uganda in 1947 from monkeys, and later in humans in 1952 [3]. After its first isolation, ZIKV was sporadically detected in Africa and Asia, however the first major outbreak was reported in Yap Island, Micronesia in 2007 [4, 5]. More recently, Zika outbreaks were reported in French Polynesia and other Pacific islands in 2013–2014 [6–8], reaching Latin America in 2013–2015 [9–11]. Zika fever was believed to cause only a mild and self-limiting illness. However, it has emerged as a new public health threat since the outbreak in French Polynesia [12] and the explosive epidemic in Brazil in 2015, when ZIKV infection was responsible for an increase in severe congenital malformations (microcephaly) and neurological complications, mainly Guillain Barré Syndrome (GBS) [13–17]. In Brazil, the virus was detected for the first time in symptomatic patients in March 2015, in the cities of Camaçari, Bahia and in Natal, Rio Grande do Norte [9], both located in northeastern Brazil. By December 2015, all regions of the country had already reported autochthonous transmission, and estimates were that Zika suspected cases ranged from 440,000 to 1,300,000 [18]. ZIKV strains are grouped into two major genotypes: African and Asian [19]; genetic analysis has revealed that the Asian genotype has been responsible for the current global expansion of the virus [5, 20, 21].

ZIKV is transmitted to humans primarily through the bite of an infected *Aedes* (*Stg*.) species mosquito, mainly *Ae*. *aegypti* and possibly by *Ae*. *albopictus* [22]. Both are exotic species in the Americas [22, 23] and took advantage of trade development to spread throughout the tropics from their native area: *Ae*. *aegypti* from Africa and *Ae*. *albopictus* from Southeast Asia. The vector transmission occurs according to the following steps: a female mosquito may become infected after taking a blood meal on a viremic individual with subsequent virus replication in the epithelium of its midgut, from where the virus may disseminates or not to secondary tissues, including the salivary glands, and finally the viral particles are available in the saliva if the insect is permissive. Then a subsequent injection of infectious saliva into a human host during a bloodmeal the transmission achieved.

Specific factors, including the mosquito and viral genetics, combined to external influences, particularly temperature, determine vector competence (VC) [24, 25]. VC is defined as the intrinsic permissiveness of a vector to infection, replication, and transmission of an agent such as a virus [26, 27].

Here, we investigated vector competence of *Ae*. *aegypti* and *Ae*. *albopictus* populations orally exposed to different infectious doses of ZIKV and incubated at two distinct temperatures aiming to better understand factors underlying a successful human-mosquito-human ZIKV transmission in Brazil.

## Materials and methods

### Ethical considerations

Mosquito-rearing protocols were approved by the Institutional Ethical Committee on Animal Use (CEUA-IOC license LW-34/14) at the Oswaldo Cruz Institute, Oswaldo Cruz Foundation. No specific permits were required to collect mosquitoes in the districts in Manaus, Natal and Rio de Janeiro.

### Mosquito populations

Four populations of Brazilian *Aedes* mosquitoes were used: (i) *Ae*. *aegypti* from Urca (AA-URC; F1 generation), Rio de Janeiro, coastal Southeast region; (ii) *Ae*. *aegypti* (AA) from Natal (AA-NAT; F1 generation), Rio Grande do Norte, coastal Northeast region; (iii) *Ae*. *albopictus* from Urca (AB-URC; F1 generation), Rio de Janeiro; (iv) *Ae*. *albopictus* from Manaus (AB-MAN; F1 generation), Amazonas, North region. The laboratory F1 mosquitoes generations were obtained from field collected eggs with ovitraps [28] settled around dwellings. After hatching, larvae were split by 150–200 individuals per pan, fed with 1 yeast tablet (LevLife, São Paulo, Brazil) renewed every 3–4 days and dissolved in 1 liter of dechlorinated tap water. Emerging adults were kept in cages at 28°C±1°C with 12:12h light-darkcycle, 80% relative humidity, and were supplied with a 10% sucrose solution.

### Viral strain

Mosquitoes were challenged with ZIKV strain of the American lineage (BRPE243/2015; GenBank KX197192), previously isolated from a patient’s blood in Pernambuco, located in the Northeast region of Brazil, during the 2015 outbreak [29]). Viral titers were quantified via plaque-forming assay prior to experimental infection. ZIKV stock was produced in Vero cells (amplification step <5) maintained with Earle’s 199 medium (Sigma Aldrich, St. Louis, MO, USA) supplemented with 5% fetal bovine serum (FBS), under an atmosphere containing 5% CO_2_, and incubated at 37°C. Viral titers were quantified via plaque-forming assay in Vero cells prior to experimental infection. ZIKV was initially amplified to a viral concentration of 10^6^ PFU/mL and later passed through a ten-fold serial dilution, producing five different viral doses, from 10^2^ to 10^6^ PFU/mL.

### Experimental ZIKV infection

Female mosquitoes at five to seven days post-emergence were isolated in feeding boxes and starved for 24 h. They were fed using an artificial feeding apparatus (Hemotek, Great Harwood, UK) with a mixture containing two parts washed erythrocytes and one part viral suspension supplemented with adenosine triphosphate (ATP) at a final concentration of 5mM. In the experimental design, for each population, 3–4 boxes of 60 mosquitoes each, per challenge dose, were exposed to the infectious blood meal, containing a total of 5 different virus doses:10^2^,10^3,^ 10^4,^ 10^5^ and 10^6^ PFU/mL. After the infectious blood meal, only fully engorged females were transferred into new containers. Half of the exposed mosquitoes were incubated at two constant incubation temperatures 28°C and 22°C,and kept at 80% of humidity under a 12:12h light-dark cycle with free access to a 10% sucrose solution.

### Mosquito infection, dissemination, and transmission potential

Mosquitoes were randomly picked at 14 and 21 days post-infection (dpi). For each population, batches of 30 mosquitoes were analyzed to estimate VC parameters. Head and body (thorax and abdomen) were individually ground in 300 μL of medium supplemented with 4% fetal bovine serum (FBS) and centrifuged at 10,000 g for 5 min at 4°C before titration. Saliva was collected from individual mosquitoes as described previously [30]. Briefly, legs and wings of each mosquito were removed followed by insertion of the proboscis into a 20 μL tip containing 5 μL FBS for 45 min. The FBS containing saliva was expelled into 45 μL serum free media, and stored at -80°C, further analysis.

Samples of body and head homogenates and saliva were serially diluted and inoculated onto monolayers of Vero cells in 96-well plates. After 1 h incubation of homogenates at 37°C, 150 μL of 2.4% CMC (carboxymethyl cellulose) in Earle’s 199 medium was added per well. Cells were incubated for 7 days at 37°C then fixed with a crystal violet solution (0.2% in 10% formaldehyde and 20% ethanol). Presence of viral particles was assessed by the detection of cytopathic effect on the cells.

Infection rate (IR) was measured as the percentage of mosquitoes with infected body (thorax and abdomen) among the total number of mosquitoes analyzed. Disseminated infection rate (DIR) was estimated as the percentage of mosquitoes with infected heads (i.e., the virus had successfully crossed the midgut barrier to reach the mosquito hemocele) among the previously detected infected mosquitoes (i.e; abdomen/thorax positive). Transmission rate (TR) represents the percentage of mosquitoes with infectious saliva among mosquitoes with disseminated infection. Transmission efficiency (TE) was calculated as the overall proportion of females with infectious saliva among the total number of mosquitoes engorged with the infectious meal.

### Statistical analysis

Statistical analysis was conducted in R environment [31]. First, overall IR, DR, TR and TE (i.e. regardless incubation temperature, virus dose and dpi) for *Ae*. *aegypti* and *Ae*. *albopictus* were compared using Pearson's Chi-squared Test for Count Data. Backward stepwise logistic regression analysis was performed to identify significant effects of mosquito population, virus titer (10^2^ to 10^6^ PFU/ml), incubation temperature (22 or 28°C), days post infection (14 or 21 dpi) (independent variables) and their interactions on mosquito infection (dependent variable). The influence of the same variables on virus dissemination in those mosquitoes with infected bodies (i.e. dissemination) and the presence of ZIKV in the saliva of mosquitoes with disseminated infection (i.e. transmission) and in the total of tested specimens (i.e. transmission efficacy) were analyzed following the same procedure. The strength of association between each independent variable and ZIKV infection/dissemination/transmission was expressed by the Odds Ratio (OR) with a 95% confidence interval (95% CI).

## Results

**Fig 1:**
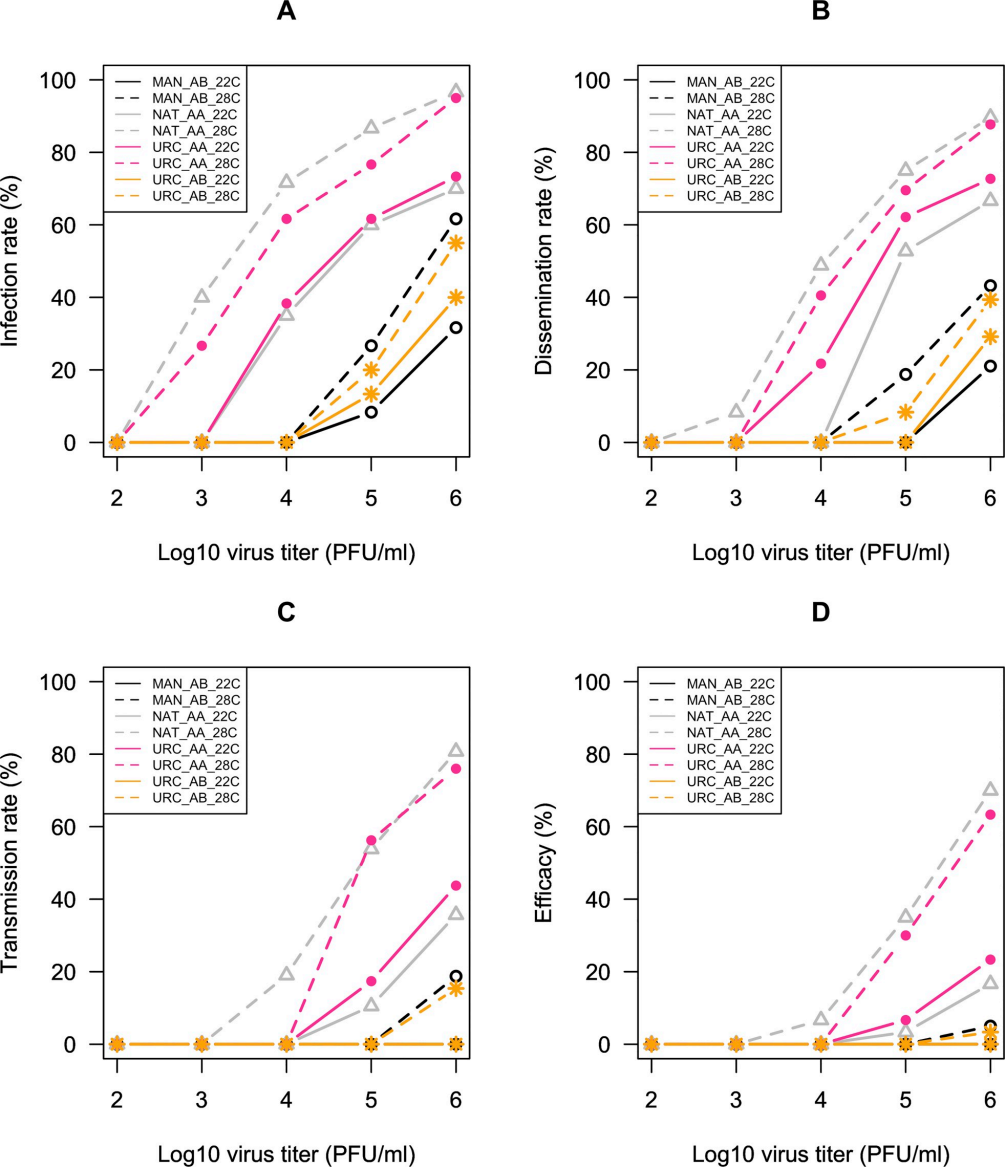
Viral infection (A), dissemination (B) and transmission (C,D) after challenge *Aedes aegypti* and *Aedes albopictus* from Brazil with five different virus dose of ZIKV, from 10^2^ to 10^6^ PFU/mL, and incubated at 22°C and 28°C.

To evaluate the effect of different components in *Aedes* vector competence, two *Aedes aegypti* populations (referred to as URC_AA and NAT_AA) and two *Aedes albopictus* populations (referred to as URC_AB and MAN_AB) from Brazil were exposed to a ZIKV infectious blood meal (titers ranging from 10^2^ to 10^6^ PFU/ml) and incubated at 22°C or 28°C. Mosquito´s body (for infection rate, IR), head (for dissemination infection rate, DR) and saliva (for transmission rate and transmission efficacy, TR and TE, respectively) were examined at 14 and 21 dpi. Since these time points exhibited low or no differences in ZIKV-positivity rates for IR, DR, TR and TE, 14 and 21 dpi data were combined in tables and graphic representations (Fig 1A–1D) to facilitate results interpretation. Raw data can be found in S1 Table.

ZIKV infection patterns were remarkable distinct between mosquito species, with overall IR, DR, TR and TE (i.e. regardless incubation temperature, virus titer and dpi) significantly higher for *Ae*. *aegypti* than for *Ae*. *albopictus* (Pearson's Chi-squared Test for Count Data for IR: χ^2^ = 295.27, df = 1, p-value < 0.01; DR: χ^2^ = 44.15, df = 1, p-value < 0.01; TR: χ^2^ = 19.75, df = 1, p-value < 0.01; TE: χ^2^ = 19.75, df = 1, p-value < 0.01) (Fig 1A–1D). Therefore, we chose to fit logistic models separately for *Ae*. *aegypti* and *Ae*. *albopictus*. Interactions between independent variables were not considered in the logistic regression analysis for *Ae*. *aegypti* TR and TE due to complete separation, as mosquitoes exhibiting virus in the saliva became relatively infrequent when data is divided in many subgroups. It was not possible to fit logistic regression models to *Ae*. *albopictus* TR and TE since only five specimens were found with positive saliva.

### ZIKV infection, dissemination and transmission in *Ae*. *aegypti*

We first analyzed the effects of mosquito population, virus titer, incubation temperature and dpi, as well as interactions among all factors, on *Ae*. *aegypti* ZIKV infection. The virus titer significantly impacted the ZIKV infection (logistic regression OR = 2.40, OR 95% CI: 1.19–4.87, z = 2.43, p-value = 0.01), with no positive mosquito feed with 10^2^ PFU/ml of virus for both populations. At 10^3^ PFU/ml, infection was only detected when mosquitoes were incubated at 28°C. The highest IRs were reported at 10^6^ PFU/ml of ZIKV: 96.7 and 95% for NAT_AA and URC_AA populations, respectively (Fig 1A, Table 1). We also found a significant correlation between IR and temperature (logistic regression OR for 28°C = 7.20, OR 95% CI: 4.48–11.59, z = 8.14, p-value < 0.01). For NAT_AA, IRs ranged from 35% (10^4^ PFU/ml) to 70% (10^6^ PFU/ml) at 22°C and from 40% (10^3^ PFU/ml) to 96.7% (10^6^ PFU/ml) at 28°C. Regarding URC_AA, IRs ranged from 38.3% (10^4^ PFU/ml) to 73.3% (10^6^ PFU/ml) at 22°C and from 26.7% (10^3^ PFU/ml) to 95% (10^6^ PFU/ml) at 28°C (Fig 1A, Table 1). The variable population was included in the final logistic regression model, but no significant difference was detected: NAT_AA and URC_AA exhibited similar IRs (46 and 43.3% regardless virus titer, temperature and dpi, respectively; S2A Table). In the same way, no statistically significant differences were detected between 14 and 21 dpi, with a slight increase in the IR from 41.7 to 47.7%, respectively (S2A Table). Finally, the IR was significantly associated with the interaction between population and temperature (logistic regression OR population:temperature = 0.52; OR 95% CI: 0.27–0.97; z = -2.04, p-value = 0.04). This suggests that *Ae*. *aegypti* populations might vary in their response to different incubation conditions: at 22°C, IR for NAT_AA and URC_AA were very similar regardless virus titers in the infectious blood meal, while, at 28°C, IR for NAT_AA were always higher than URC_AA, except when fed with 10^6^ PFU/ml of ZIKV (Fig 1A, Table 1).

**Table 1:** *Aedes aegypti* infection, dissemination, transmission and transmission efficacy according to mosquito population, ZIKV titer and incubation temperature.

Population	Virus titer (PFU/ml) *	Incubation T°C	Infection rate, %	Dissemination rate, %	Transmission rate, %	Transmission efficacy, %
NAT_AA	10^4^	22°C	35 (21/60)	0 (0/21)	-	0 (0/60)
	10^5^		60 (36/60)	52.78 (19/36)	10.53 (2/19)	3.33 (2/60)
	10^6^		70 (42/60)	66.67 (28/42)	35.71 (10/28)	16.67 (10/60)
	10^3^	28°C	40 (24/60)	8.33 (2/24)	0 (0/2)	0 (0/60)
	10^4^		71.67 (43/60)	48.84 (21/43)	19.05 (4/21)	6.67 (4/60)
	10^5^		86.67 (52/60)	75 (39/52)	53.85 (21/52)	35 (21/60)
	10^6^		96.67 (58/60)	89.66 (52/58)	80.77 (42/52)	70 (42/60)
URC_AA	10^4^	22°C	38.33 (23/60)	21.74 (5/23)	0 (0/5)	0 (0/60)
	10^5^		61.67 (37/60)	62.16 (23/37)	17.39 (4/23)	6.67 (4/60)
	10^6^		73.33 (44/60)	72.73 (32/44)	43.75 (14/32)	23.33 (14/60)
	10^3^	28°C	26.67 (16/60)	0 (0/16)	-	0 (0/60)
	10^4^		61.67 (37/60)	40.54 (15/37)	0 (0/15)	0 (0/60)
	10^5^		76.67 (46/60)	69.57 (32/46)	56.25 (18/32)	30 (18/60)
	10^6^		95 (57/60)	80.72 (50/57)	76 (38/57)	63.33 (38/60)

14 and 21 dpi data were combined since they exhibited low or no differences in ZIKV-positivity rates.

* Virus titer data showing no ZIKV infected mosquitoes was omitted. Raw complete data can be found in S1 Table.

After, we analyzed the effects of the same variables on the probability of infected *Ae*. *aegypti* mosquitoes presenting disseminated ZIKV infection. Virus titer in the infectious meal and temperature were significantly correlated with the DR (logistic regression OR for virus titer = 3.84; OR 95% CI: 2.99–4.93; z = 10.52, p-value < 0.01; OR for 28°C = 4.47; OR 95% CI: 2.44–8.20; z = 4.84, p-value < 0.01). When taking 10^3^PFU/ml of ZIKV, viral dissemination was registered only for NAT_AA population incubated at 28°C. From 10^4^ PFU/ml onwards, DR was always higher at 28°C and increased with virus titer for both populations, reaching up to 89.6% for NAT_AA (Fig 1B, Table 1). Although populations exhibited similar overall DRs (58.3 and 60.4% for NAT_AA and URC_AA, respectively, disregarding the different incubation temperatures and virus titers in the infectious meal, S2B Table), they behaved differently when maintained at 22 or 28°C (logistic regression OR population:temperature = 0.43; OR 95% CI: 0.19–0.98; z = -1.10, p-value = 0.045). DRs for URC_AA samples were more homogeneous between 22 and 28°C than the NAT_AA samples under the same incubation conditions (Fig 1B, Table 1). The dpi (14 and 21 days) did not influence dissemination.

Considering the TR, the variables "virus titer" and "temperature" were included in the final logistic regression model, both being significant (logistic regression OR for 28°C = 6.15; OR 95% CI: 3.45–10.95; z = 6.17, p-value < 0.01). Overall, TRs were similar for NAT_AA and URC_AA populations (49.1 and 47.1%, respectively) and 14 and 21 dpi (46.4 and 49.4%, respectively) (S2C Table). ZIKV transmission was possible from the ingestion of blood holding at least 10^5^ PFU/ml of virus particles at both incubation temperatures, except for the NAT_AA incubated at 28°C, which exhibited saliva-positive mosquitoes that had taken meals containing from 10^4^ PFU/ml of ZIKV onwards. At 22°C, TR ranged from 10.5% (NAT_AA at 10^5^ PFU/ml of ZIKV) and 43.7% (URC_AA at 10^6^ PFU/ml of ZIKV), while it was between 19% (NAT_AA at 10^4^ PFU/ml of ZIKV) and 80.8% (NAT_AA at 10^6^ PFU/ml of ZIKV) at 28°C (Fig 1C, Table 1). Epidemiologically, the percentage of mosquitoes that are able to deliver infectious virus in their saliva among all specimens exposed to an infectious blood meal (i.e. the vector competence) is the most important phenotype and can be adequately measured by the TE. We found a significant association between TE and virus titer, temperature and dpi for *Ae*. *aegypti* (logistic regression OR for virus titer = 6.97, OR 95% CI: 5.02–9.69, z = 11.576; p-value < 0.01; OR for 28°C = 15.27, OR 95% CI: 7.27–32.07, z = 7.20; p-value < 0.01; OR for 21dpi = 1.09, OR 95% CI: 1.02–1.16, z = 2.57; p-value = 0.01). There was a remarkable increase in ZIKV saliva-positive mosquitoes with virus titer in the infectious meal, i.e. from 6.7% (NAT_AA, 28°C) at 10^4^ PFU/ml to 70% (NAT_AA, 28°C) at 10^6^ PFU/ml of virus. TE was also higher for both *Ae*. *aegypti* populations incubated at 28°C than at 22°C at all virus titers (Fig 1D, Table 1). Finally, we registered a slightly increase in the TE with the time post infection, as 10.8% of *Ae*. *aegypti* were saliva-positive for ZIKV after 14 dpi against 14.7% at 21 dpi (combining populations, incubation temperatures and virus doses, S2D Table).

### ZIKV infection, dissemination and transmission in *Ae*. *albopictus*

We analyzed the effects of mosquito population, virus titer, incubation temperature and dpi, as well as interactions among all factors, on *Ae*. *albopictus* ZIKV IR. Among all tested variables, virus titer in the infectious meal (logistic regression OR = 7.61; OR 95% CI: 5.42–10.67, z = 11.74, p-value < 0.01) and incubation temperature were those significantly impacting IR (OR = 4.21; OR 95% CI: 2.04–7.04, z = 11.74, p-value < 0.01). *Ae*. *albopictus* only became infected when taking 10^5^ and 10^6^ PFU/ml of ZIKV regardless the incubation temperature (S3A Table). At 22°C, IRs ranged from 8.3% (MAN_AB, 10^5^ PFU/ml) to 40% (URC_AB, 10^6^ PFU/ml), while at 28°C they varied between 20% (URC_AB, 10^5^ PFU/ml) and 61.7% (MAN_AB, 10^6^ PFU/ml) (Fig 1A, Table 2). Virus titer and temperature also significantly impacted ZIKV dissemination (logistic regression OR for virus titer = 5.52, OR 95% CI: 1.81–16.80, z = 3.01, p-value < 0.01; OR for 28°C = 2.34, OR 95% CI: 1.05–5.24, z = 2.07, p-value = 0.04) (S3B Table). At 22°C, dissemination was only possible at 10^6^ PFU/ml of virus with DRs between 21.2 and 29.2% for MAN_AB and URC_AB, respectively. At 28°C, *Ae*. *albopictus* DRs were 8.3 and 39.4% (URC_AB) and 18.7 and 43.2% (MAN_AB) at 10^5^ and 10^6^ PFU/ml, respectively (Fig 1B, Table 2). Regarding transmission, ZIKV was detected in the saliva of five *Ae*. *albopictus* females, three from the MAN_AB and two from the URC_AB, all of them at 10^6^ PFU/ml of virus at 28°C. Under these conditions, TR and TE were 15.4 and 3.3%, respectively, for the MAN_AB and 18.7 and 5%, respectively, for the URC_AB (Fig 1C and 1D, Tables 2 and S3C and S3D).

**Table 2:** *Aedes albopictus* infection, dissemination, transmission and transmission efficacy according to mosquito population, ZIKV titer and incubation temperature.

Population	Virus titer (PFU/ml) ^a^	Incubation T°C	Infection rate, %	Dissemination rate, %	Transmission rate, %	Transmission efficacy, %
URC_AB	10^5^	22°C	13.33 (8/60)	0 (0/8)	-	0 (0/60)
	10^6^		40 (24/60)	29.17 (7/24)	0 (0/7)	0 (0/60)
	10^5^	28°C	20 (12/60)	8.33 (1/12)	0 (0/1)	0 (0/60)
	10^6^		55 (33/60)	39.39 (13/33)	15.38 (2/13)	3.33 (2/60)
MAN_AB	10^5^	22°C	8.33 (5/60)	0 (0/5)	-	0 (0/60)
	10^6^		31.67 (19/60)	21.25 (4/19)	0 (0/4)	0 (0/60)
	10^5^	28°C	26.67 (16/60)	18.75 (3/16)	0 (0/3)	0 (0/60)
	10^6^		61.67 (37/60)	43.24 (16/37)	18.75 (3/16)	5 (3/60)

14 and 21 dpi data were combined since they exhibited low or no differences in ZIKV-positivity rates. * Virus titer data showing no ZIKV infected mosquitoes was omitted. Raw complete data can be found in S1 Table.

## Discussion

In this study, we provide evidence that the transmission potential of ZIKV by *Aedes aegypti* and *Aedes albopictus* depends on a complex interaction between mosquito vector population, temperature and viral infectious dose in the blood meal. Generally high viral doses are used in experimental studies, which probably do not occur in nature, as ZIKV blood viremia has been shown to be on average lower than observed in other arbovirus systems [5, 32]. Understanding mosquito infectivity at different viremia levels is important in assessing the role of virus titer capable of successfully sustaining human-to-mosquito ZIKV transmission. In addition, further investigations of genetic and environmental contributions are needed, such as the interactions between mosquito population, viral strain and temperature on the viral transmission potential, which is still poorly explored for ZIKV.

Our results indicated that the viremia in the blood meal had an effect on probability of *Aedes* mosquitoes becoming infected, disseminating infection and subsequently expectorating viral particles. Comparatively, at a temperature of 28°C, the lowest dose of virus in artificial blood meals required for viral infection and dissemination in *Ae*. *albopictus* was 10^5^ PFU/mL. However, in *Ae*. *aegypti*, smaller virus doses were required for virus infection: 10^3^ PFU/mL for Natal population and 10^4^ PFU/mL for Rio de Janeiro population, while for the virus dissemination was necessary a virus titer of 10^4^ PFU/mL for both populations. It is worth noting that *Ae*. *aegypti* population from Natal when challenged with the different virus doses showed higher IR, DIR and TR when compared to the *Ae*. *aegypti* population from Rio de Janeiro, at both temperatures. Maybe, this outcome is due to a close interaction between the vector population and the viral genotype, both originating from Northeastern Brazil. These variations demonstrate the importance of considering genetic variation of populations when assessing vector competence.

Roundy *et al* 2017 [33] revealed variation in vector competence of *Ae*. *aegypti* from Salvador/Brazil when orally exposed to ZIKV strain from Mexico (Asian genotype) with different virus doses (10^4^,10^5^ e 10^6^ FFU/mL) at 27° ± 1°C. According to these authors, high infection and dissemination only occurred after the challenged *Aedes* population from Salvador took artificial blood meals with a concentration of 10^6^ FFU/mL and showed no dissemination with a concentration of 10^4^ FFU/mL at 14 dpi. Ciota *et al* 2017 [34] suggested that the minimum infective dose of ZIKV to *Aedes* is 10^4.2^ PFU/mL at 27°C, while infection was detected in the two *Ae*. *aegypti* populations we orally challenged with 10^2^ and 10^3^ PFU/mL and incubated at a temperature of only 1°C less (27°C). Our findings show that rates of infection and dissemination in *Ae*.*aegypti* were significantly reduced when artificial blood meal titers were less than 10^4^ PFU/mL.

*Ae*. *aegypti* and *Ae*. *albopictus* were unable to transmit ZIKV with a virus doses of 10^2^ and 10^3^ PFU/mL despite the incubation temperature (22°C and 28°C). *Ae*. *aegypti* showed higher TE when taking a virus doses of 10^5^ and 10^6^ PFU/mL at 28°C, while both *Ae*. *albopictus* populations presented a null transmission efficiency of the ZIKV incubated at 22°C and significantly low TE even at 28°C, even taking a blood containing the highest viral titers, such as 10^5^ and 10 ^6^ PFU/mL. Azar *et al*., 2017 [35] showed that vector competence in *Ae*. *albopictus* is potentially dependent on geographic origin of both the mosquito population and the viral strain. An *Ae*. *albopictus* population from Salvador, northeast Brazil, tested by these authors shed no virus into saliva in 14 days of extrinsic incubation at 27 ± 1°C even when orally exposed to high titers (6 or 7 log10 FFU/mL) of two American strains of ZIKV.

The statistical differences in infection and transmission rates among these species suggest the presence of a midgut barrier to dissemination and, more significantly, a strong salivary gland barrier in *Ae*. *albopictus*. Overcoming such barriers occurred only after the challenged *Ae*. *albopictus* population took artificial blood meals with a concentration of 10^5^ PFU/mL at 28°C.

However, it is important to mention that despite the limited vector capacity showed by *Ae*. *albopictus*, at least for the ZIKV strain circulating in Brazil, adaptive mutations may occur over time leading to an increase in ZIKV transmission efficiency, as described for CHIKV[36]. This finding highlights the need to consider the complex interplay between genetic and environmental variabilities for better understanding of pathogen-host interactions.

We also show that the effects of variation in virus dose on vector competence is strongly driven by temperature. In other arbovirus systems, studies have already demonstrated that temperature may alter interactions between the virus genotype and the mosquito genotype, affecting significantly the vector competence [37–44]. Interestingly, we found that the relationship between IR, DIR and TR changed depending on the temperature of incubation. For ZIKV, we demonstrated that *Ae*. *aegypti* and *Ae*. *albopictus* populations from Brazil presented higher TRs at 28°C than at 22°C with the same virus doses, indicating that virus transmission was significantly determined by incubation temperature. According to Tesla *et al*. 2018 low temperatures restrict midgut escape and dissemination, resulting in a lower proportion of the mosquito population that become infectious. Warmer temperatures, on the other hand, were very permissive for ZIKV infection with ZIKV transmission optimized at a mean temperature of approximately 29°C. Daily temperature fluctuations that occur under natural environmental conditions have been shown to influence the vector competence in dengue viruses [45]. Although the effect of temperature on vector competence has been assessed using constant temperatures, our results indicate that seasonal temperature variation in Brazil would likely affect ZIKV replication within *Aedes* populations.

Notably, *Aedes aegypti* populations used in this study efficiently transmitted ZIKV after orally challenged with a virus dose of 10^6^ PFU/mL and incubated at 28°C, showing high rates of infection, dissemination and transmission, according with Fernandes *et al*. 2016 that described high vector competence in several Brazilian populations of *Ae*. *aegypti* strains challenged with three strains of ZIKV also isolated in Brazil [46, 47]. There is a strong evidence that vector competence can vary across mosquito populations due the specific combination of mosquito and ZIKV, dengue and chikungunya virus genotypes [24, 25, 33, 39, 48–50]. We hypothesized that higher transmission rates could be due to the fact that we paired ZIKV belong to the American lineage that was previously isolated from human from the city of Recife, northeast Brazil [29] and *Aedes aegypti* and *Aedes albopictus* population collected in the same country.

In conclusion, we experimentally demonstrated that the tested Brazilian populations of *Ae*. *aegypti* exhibit a higher transmission potential for ZIKV than *Ae*. *albopictus*, but the virus dose and temperature were significant predictors of the proportion of mosquito whose bites became infectious. Combined, our results indirectly reinforce the main role of *Ae*. *aegypti* in ZIKV transmission in Brazil.

## Supporting information

S1 Table Infection, dissemination, transmission and transmission efficacy for *Aedes aegypti* and *Aedes albopictus* according to mosquito population, ZIKV dose, incubation temperature and days post infection. (DOC)

S2 Table Backward stepwise logistic regression analysis to evaluate the influence of mosquito population, incubation temperature, virus titer and days post infection on *Aedes aegypti* ZIKV infection (A), dissemination (B), transmission (C) and transmission efficacy (D) rates.
(DOC)

S3 Table Backward stepwise logistic regression analysis to evaluate the influence of mosquito population, incubation temperature, virus titer and days post infection on *Aedes albopictus* ZIKV infection (A), dissemination (B), transmission (C) and transmission efficacy (D) rates.
(DOC)
