# Supplementary material for: Zika virus transmission by Brazilian Aedes aegypti and Aedes albopictus is virus dose and temperature-dependent
Source: PLoS Negl Trop Dis. 2020 Sep 8;14(9):e0008527. doi: 10.1371/journal.pntd.0008527 (PMC7500593; doi:10.1371/journal.pntd.0008527)
Supplement: S1 Table — (DOC) [file pntd.0008527.s001.doc]

**Supplementary Information**

**Table S1.** Infection, dissemination, transmission and transmission efficacy for *Aedes aegypti* and *Aedes albopictus* according to mosquito population, ZIKV dose, incubation temperature and days post infection.

| **Mosquito species** | **Population** | **Virus titer (PFU/ml)** | **Incubation T°C** | **Days post infection** | **Infection rate, %** | **Dissemination rate, %** | **Transmission rate, %** | **Transmission efficacy, %** |
| --- | --- | --- | --- | --- | --- | --- | --- | --- |
| *Aedes aegypti* | URC_AA | 10^2^ | 22ºC | 14 | 0 (0/30) | - | - | 0 (0/30) |
|  |  | 10^3^ |  |  | 0 (0/30) | - | - | 0 (0/30) |
|  |  | 10^4^ |  |  | 36.67 (11/30) | 18.18 (2/11) | 0 (0/2) | 0 (0/30) |
|  |  | 10^5^ |  |  | 53.33 (16/30) | 56.25 (9/16) | 0 (0/9) | 0 (0/30) |
|  |  | 10^6^ |  |  | 70 (21/30) | 71.43 (15/21) | 40 (6/15) | 20 (6/30) |
|  | URC_AA | 10^2^ | 22ºC | 21 | 0 (0/30) | - | - | 0 (0/30) |
|  |  | 10^3^ |  |  | 0 (0/30) | - | - | 0 (0/30) |
|  |  | 10^4^ |  |  | 40 (12/30) | 25 (3/12) | 0 (0/3) | 0 (0/30) |
|  |  | 10^5^ |  |  | 70 (21/30) | 66.67 (14/21) | 28.47 (4/14) | 13.33 (4/30) |
|  |  | 10^6^ |  |  | 76.67 (23/30) | 73.91 (17/23) | 47.06 (8/17) | 26.67 (8/30) |
|  | URC_AA | 10^2^ | 28ºC | 14 | 0 (0/30) | - | - | 0 (0/30) |
|  |  | 10^3^ |  |  | 23.33 (7/30) | 0 (0/7) | - | 0 (0/30) |
|  |  | 10^4^ |  |  | 56.67 (17/30) | 35.29 (6/17) | 0 (0/6) | 0 (0/30) |
|  |  | 10^5^ |  |  | 73.33 (22/30) | 68.18 (15/22) | 53.33 (8/15) | 26.67 (8/30) |
|  |  | 10^6^ |  |  | 90 (27/30) | 85.19 (23/27) | 78.26 (18/23) | 60 (18/30) |
|  | URC_AA | 10^2^ | 28ºC | 21 | 0 (0/30) | - | - | 0 (0/30) |
|  |  | 10^3^ |  |  | 30 (9/30) | 0 (0/9) | - | 0 (0/30) |
|  |  | 10^4^ |  |  | 66.67 (20/30) | 45 (9/20) | 0 (0/9) | 0 (0/30) |
|  |  | 10^5^ |  |  | 80 (24/30) | 70.83 (17/24) | 58.82 (10/17) | 33.33 (10/30) |
|  |  | 10^6^ |  |  | 100 (30/30) | 90 (27/30) | 74.04 (20/27) | 66.67 (20/30) |
|  | NAT_AA | 10^2^ | 22ºC | 14 | 0 (0/30) | - | - | 0 (0/30) |
|  |  | 10^3^ |  |  | 0 (0/30) | - | - | 0 (0/30) |
|  |  | 10^4^ |  |  | 33.33 (10/30) | 0 (0/10) | - | 0 (0/30) |
|  |  | 10^5^ |  |  | 53.33 (16/30) | 43.75 (7/16) | 0 (0/7) | 0 (0/30) |
|  |  | 10^6^ |  |  | 66.67 (20/30) | 65 (13/20) | 30.77 (4/13) | 13.33 (4/30) |
|  | NAT_AA | 10^2^ | 22ºC | 21 | 0 (0/30) | - | - | 0 (0/30) |
|  |  | 10^3^ |  |  | 0 (0/30) | - | - | 0 (0/30) |
|  |  | 10^4^ |  |  | 36.67 (11/30) | 0 (0/11) | - | 0 (0/30) |
|  |  | 10^5^ |  |  | 66.67 (20/30) | 60 (12/20) | 16.67 (2/12) | 6.67 (2/30) |
|  |  | 10^6^ |  |  | 73.33 (22/30) | 68.18 (15/22) | 40 (6/15) | 20 (6/30) |
|  | NAT_AA | 10^2^ | 28ºC | 14 | 0 (0/30) | - | - | 0 (0/30) |
|  |  | 10^3^ |  |  | 36.67 (11/30) | 0 (0/11) | - | 0 (0/30) |
|  |  | 10^4^ |  |  | 66.67 (20/30) | 45 (9/20) | 0 (0/9) | 0 (0/30) |
|  |  | 10^5^ |  |  | 80 (24/30) | 70.83 (17/24) | 52.94 (9/17) | 30 (9/30) |
|  |  | 10^6^ |  |  | 93.33 (28/30) | 85.71 (24/28) | 83.33 (20/24) | 66.67 (20/30) |
|  | NAT_AA | 10^2^ | 28ºC | 21 | 0 (0/30) | - | - | 0 (0/30) |
|  |  | 10^3^ |  |  | 43.33 (13/30) | 15.38 (2/13) | 0 (0/2) | 0 (0/30) |
|  |  | 10^4^ |  |  | 76.67 (23/30) | 52.17 (12/23) | 33.33 (4/12) | 13.33 (4/30) |
|  |  | 10^5^ |  |  | 93.33 (28/30) | 78.57 (22/28) | 54.55 (12/22) | 40 (12/30) |
|  |  | 10^6^ |  |  | 100 (30/30) | 93.33 (28/30) | 78.57 (22/28) | 73.33 (22/30) |
| *Aedes albopictus* | URC_AB | 10^2^ | 22ºC | 14 | 0 (0/30) | - | - | 0 (0/30) |
|  |  | 10^3^ |  |  | 0 (0/30) | - | - | 0 (0/30) |
|  |  | 10^4^ |  |  | 0 (0/30) | - | - | 0 (0/30) |
|  |  | 10^5^ |  |  | 10 (3/30) | 0 (0/3) | - | 0 (0/30) |
|  |  | 10^6^ |  |  | 36.67 (11/30) | 27.27 (3/11) | 0 (0/3) | 0 (0/30) |
|  | URC_AB | 10^2^ | 22ºC | 21 | 0 (0/30) | - | - | 0 (0/30) |
|  |  | 10^3^ |  |  | 0 (0/30) | - | - | 0 (0/30) |
|  |  | 10^4^ |  |  | 0 (0/30) | - | - | 0 (0/30) |
|  |  | 10^5^ |  |  | 16.47 (5/30) | 0 (0/5) | - | 0 (0/30) |
|  |  | 10^6^ |  |  | 43.33 (13/30) | 30.77 (4/13) | 0 (0/4) | 0 (0/30) |
|  | URC_AB | 10^2^ | 28ºC | 14 | 0 (0/30) | - | - | 0 (0/30) |
|  |  | 10^3^ |  |  | 0 (0/30) | - | - | 0 (0/30) |
|  |  | 10^4^ |  |  | 0 (0/30) | - | - | 0 (0/30) |
|  |  | 10^5^ |  |  | 16.67 (5/30) | 0 (0/5) | - | 0 (0/30) |
|  |  | 10^6^ |  |  | 53.33 (16/30) | 37.5 (6/16) | 16.67 (1/6) | 3.33 (1/30) |
|  | URC_AB | 10^2^ | 28ºC | 21 | 0 (0/30) | - | - | 0 (0/30) |
|  |  | 10^3^ |  |  | 0 (0/30) | - | - | 0 (0/30) |
|  |  | 10^4^ |  |  | 0 (0/30) | - | - | 0 (0/30) |
|  |  | 10^5^ |  |  | 23.33 (7/30) | 14.29 (1/7) | 0 (0/1) | 0 (0/30) |
|  |  | 10^6^ |  |  | 56.67 (17/30) | 41.18 (7/17) | 14.29 (1/7) | 3.33 (1/30) |
|  | MAN_AB | 10^2^ | 22ºC | 14 | 0 (0/30) | - | - | 0 (0/30) |
|  |  | 10^3^ |  |  | 0 (0/30) | - | - | 0 (0/30) |
|  |  | 10^4^ |  |  | 0 (0/30) | - | - | 0 (0/30) |
|  |  | 10^5^ |  |  | 6.67 (2/30) | 0 (0/2) | - | (0/30) |
|  |  | 10^6^ |  |  | 30 (9/30) | 22.22 (2/9) | 0 (0/2) | (0/30) |
|  | MAN_AB | 10^2^ | 22ºC | 21 | 0 (0/30) | - | - | 0 (0/30) |
|  |  | 10^3^ |  |  | 0 (0/30) | - | - | 0 (0/30) |
|  |  | 10^4^ |  |  | 0 (0/30) | - | - | 0 (0/30) |
|  |  | 10^5^ |  |  | 10 (3/30) | 0 (0/3) | - | 0 (0/30) |
|  |  | 10^6^ |  |  | 33.3 (10/30) | 20 (2/10) | 0 (0/2) | (0/30) |
|  | MAN_AB | 10^2^ | 28ºC | 14 | 0 (0/30) | - | - | 0 (0/30) |
|  |  | 10^3^ |  |  | 0 (0/30) | - | - | 0 (0/30) |
|  |  | 10^4^ |  |  | 0 (0/30) | - | - | 0 (0/30) |
|  |  | 10^5^ |  |  | 26.67 (8/30) | 12.5 (1/8) | 0 (0/1) | 0 (0/30) |
|  |  | 10^6^ |  |  | 56.67 (17/30) | 41.18 (7/17) | 14.29 (1/7) | 3.33 (1/30) |
|  | MAN_AB | 10^2^ | 28ºC | 21 | 0 (0/30) | - | - | 0 (0/30) |
|  |  | 10^3^ |  |  | 0 (0/30) | - | - | 0 (0/30) |
|  |  | 10^4^ |  |  | 0 (0/30) | - | - | 0 (0/30) |
|  |  | 10^5^ |  |  | 26.67 (8/30) | 25 (2/8) | 0 (0/2) | 0 (0/30) |
|  |  | 10^6^ |  |  | 66.67 (20/30) | 45 (9/20) | 22.22 (2/9) | 6.67 (2/30) |
