# Supplementary material for: Zika virus transmission by Brazilian Aedes aegypti and Aedes albopictus is virus dose and temperature-dependent
Source: PLoS Negl Trop Dis. 2020 Sep 8;14(9):e0008527. doi: 10.1371/journal.pntd.0008527 (PMC7500593; doi:10.1371/journal.pntd.0008527)
Supplement: S2 Table — Backward stepwise logistic regression analysis to evaluate the influence of mosquito population, incubation temperature, virus titer and days post infection on Aedes aegypti ZIKV infection (A), dissemination (B), transmission (C) and transmission efficacy (D) rates. (DOC) [file pntd.0008527.s002.doc]

**Supplementary Information**

**Table S2.** Backward stepwise logistic regression analysis to evaluate the influence of mosquito population, incubation temperature, virus titer and days post infection on *Aedes aegypti* ZIKV infection (A), dissemination (B), transmission (C) and transmission efficacy (D) rates.

A) Final logistic model: infection ~ population + temperature + virus titer + day post infection + population:temperature + virus titer:day post infection.

|  | **ZIKV infection** | | | **Multiple logistic regression** | | | | |
| --- | --- | --- | --- | --- | --- | --- | --- | --- |
| **Variables** | **Negative (%)** | **Positive (%)** | **Total** | **z-statistic** | **p-value** | **OR** | **Lower 95% CI** | **Upper 95% CI** |
| **Population** |  |  |  | 0.57 | 0.57 | 1.14 | 0.73 | 1.79 |
| NAT_AA | 324 (54) | 276 (46) | 600 |  |  |  |  |  |
| URC_AA | 340 (56.7) | 260 (43.3) | 600 |  |  |  |  |  |
| **Temperature** |  |  |  | 8.14 | < 0.01 | 7.20 | 4.48 | 11.59 |
| 22ºC | 397 (66.2) | 203 (33.8) | 600 |  |  |  |  |  |
| 28ºC | 267 (44.5) | 333 (55.5) | 600 |  |  |  |  |  |
| **Virus titer** |  |  |  | 2.43 | 0.01 | 2.40 | 1.19 | 4.87 |
| 10^2^ | 240 (100) | 0 (0) | 240 |  |  |  |  |  |
| 10^3^ | 200 (83.3) | 40 (16.7) | 240 |  |  |  |  |  |
| 10^4^ | 116 (48.3) | 124 (51.7) | 240 |  |  |  |  |  |
| 10^5^ | 69 (28.7) | 171 (71.3) | 240 |  |  |  |  |  |
| 10^6^ | 39 (16.2) | 201 (83.8) | 240 |  |  |  |  |  |
| **Day post infection** |  |  |  | -0.74 | 0.46 | 0.94 | 0.78 | 1.11 |
| 14 dpi | 350 (58.3) | 250 (41.7) | 600 |  |  |  |  |  |
| 21 dpi | 314 (52.3) | 286 (47.7) | 600 |  |  |  |  |  |
| **Population:temperature** |  |  |  | -2.04 | 0.04 | 0.52 | 0.27 | 0.97 |
| **Virus titer:day post infection** |  |  |  | 1.52 | 0.13 | 1.03 | 0.99 | 1.07 |

B) Final logistic model: dissemination ~ population + temperature + virus titer + day post infection + population:temperature.

|  | **ZIKV dissemination** | | | **Multiple logistic regression** | | | | | |
| --- | --- | --- | --- | --- | --- | --- | --- | --- | --- |
| **Variables** | **Negative (%)** | **Positive (%)** | **Total** | **z-statistic** | **p-value** | **OR** | **Lower 95% CI** | | **Upper 95% CI** |
| **Population** |  |  |  | 1.67 | 0.09 | 1.70 | 0.91 | | 3.18 |
| NAT_AA | 115 (41.7) | 161 (58.3) | 276 |  |  |  |  | |  |
| URC_AA | 103 (39.6) | 157 (60.4) | 260 |  |  |  |  | |  |
| **Temperature** |  |  |  | 4.84 | < 0.01 | 4.47 | 2.44 | | 8.20 |
| 22ºC | 96 (47.3) | 107 (52.7) | 203 |  |  |  |  | |  |
| 28ºC | 122 (36.6) | 211 (63.4) | 333 |  |  |  |  | |  |
| **Virus titer** |  |  |  | 10.52 | < 0.01 | 3.84 | 2.99 | | 4.93 |
| 10^2^ | 0 (0) | 0 (0) | 0 |  |  |  |  | |  |
| 10^3^ | 38 (95) | 2 (5) | 40 |  |  |  |  | |  |
| 10^4^ | 83 (66.9) | 41 (33.1) | 124 |  |  |  |  | |  |
| 10^5^ | 58 (33.9) | 113 (66.1) | 171 |  |  |  |  | |  |
| 10^6^ | 39 (19.4) | 162 (80.6) | 201 |  |  |  |  | |  |
| **Day post infection** |  |  |  | 1.92 | 0.05 | 1.06 | 1.00 | 1.12 | |
| 14 dpi | 110 (44) | 140 (56) | 250 |  |  |  |  | |  |
| 21 dpi | 108 (37.8) | 178 (62.2) | 286 |  |  |  |  | |  |
| **Population:temperature** |  |  |  | -1.10 | 0.045 | 0.43 | 0.19 | | 0.98 |

C) Final logistic model: transmission ~ population + temperature + virus titer + day post infection.

|  | **ZIKV transmission** | | | **Multiple logistic regression*^1^** | | | | |
| --- | --- | --- | --- | --- | --- | --- | --- | --- |
| **Variables** | **Negative (%)** | **Positive (%)** | **Total** | **z-statistic** | **p-value** | **OR** | **Lower 95% CI** | **Upper 95% CI** |
| **Population** |  |  |  | not included in the final logistic model | | | | |
| NAT_AA | 82 (50.9) | 79 (49.1) | 161 |  |  |  |  |  |
| URC_AA | 83 (52.9) | 74 (47.1) | 157 |  |  |  |  |  |
| **Temperature** |  |  |  | 6.17 | < 0.01 | 6.15 | 3.45 | 10.95 |
| 22ºC | 77 (72.0) | 30 (28.0) | 107 |  |  |  |  |  |
| 28ºC | 88 (41.7) | 123 (58.2) | 211 |  |  |  |  |  |
| **Virus titer** |  |  |  | 7.11 | < 0.01 | 4.46 | 2.95 | 6.74 |
| 10^2^ | 0 (0) | (0) | 0 |  |  |  |  |  |
| 10^3^ | 2 (100) | 0 (0) | 2 |  |  |  |  |  |
| 10^4^ | 37 (90.2) | 4 (9.8) | 41 |  |  |  |  |  |
| 10^5^ | 68 (60.2) | 45 (39.8) | 113 |  |  |  |  |  |
| 10^6^ | 58 (35.8) | 104 (64.2) | 162 |  |  |  |  |  |
| **Day post infection** |  |  |  | not included in the final logistic model | | | | |
| 14 dpi | 75 (53.6) | 65 (46.4) | 140 |  |  |  |  |  |
| 21 dpi | 90 (50.6) | 88 (49.4) | 178 |  |  |  |  |  |

D) Final logistic model: transmission efficacy ~ population + temperature + virus titer + day post infection + population:temperature.

|  | **ZIKV transmission efficacy** | | | **Multiple logistic regression*^2^** | | | | |
| --- | --- | --- | --- | --- | --- | --- | --- | --- |
| **Variables** | **Negative (%)** | **Positive (%)** | **Total** | **z-statistic** | **p-value** | **OR** | **Lower 95% CI** | **Upper 95% CI** |
| **Population** |  |  |  | 1.21 | 0.23 | 1.64 | 0.73 | 3.68 |
| NAT_AA | 521 (86.8) | 79 (13.2) | 600 |  |  |  |  |  |
| URC_AA | 526 (87.7) | 74 (12.3) | 600 |  |  |  |  |  |
| **Temperature** |  |  |  | 7.20 | < 0.01 | 15.27 | 7.27 | 32.07 |
| 22ºC | 570 (95.0) | 30 (5.0) | 600 |  |  |  |  |  |
| 28ºC | 477 (79.5) | 123 (20.5) | 600 |  |  |  |  |  |
| **Virus titer** |  |  |  | 11.576 | < 0.01 | 6.97 | 5.02 | 9.69 |
| 10^2^ | 240 (100) | 0 (0) | 240 |  |  |  |  |  |
| 10^3^ | 240 (100) | 0 (0) | 240 |  |  |  |  |  |
| 10^4^ | 236 (98.3) | 4 (1.7) | 240 |  |  |  |  |  |
| 10^5^ | 195 (81.2) | 45 (18.8) | 240 |  |  |  |  |  |
| 10^6^ | 136 (56.7) | 104 (43.3) | 240 |  |  |  |  |  |
| **Day post infection** |  |  |  | 2.57 | 0.01 | 1.09 | 1.02 | 1.16 |
| 14 dpi | 535 (89.2) | 65 (10.8) | 600 |  |  |  |  |  |
| 21 dpi | 512 (85.3) | 88 (14.7) | 600 |  |  |  |  |  |
| **Population:temperature** |  |  |  | -1.83 | 0.07 | 0.40 | 0.15 | 1.07 |

OR= odds ratio, CI = confidence interval.

*1: logistic regression was adjusted without interactions due to complete separation.

*2: logistic regression was adjusted only with the interaction between population and temperature, due to complete separation.
