# Supplementary material for: Zika virus transmission by Brazilian Aedes aegypti and Aedes albopictus is virus dose and temperature-dependent
Source: PLoS Negl Trop Dis. 2020 Sep 8;14(9):e0008527. doi: 10.1371/journal.pntd.0008527 (PMC7500593; doi:10.1371/journal.pntd.0008527)
Supplement: S3 Table — Backward stepwise logistic regression analysis to evaluate the influence of mosquito population, incubation temperature, virus titer and days post infection on Aedes albopictus ZIKV infection (A), dissemination (B), transmission (C) and transmission efficacy (D) rates. (DOC) [file pntd.0008527.s003.doc]

**Supplementary Information**

**Table S3.** Backward stepwise logistic regression analysis to evaluate the influence of mosquito population, incubation temperature, virus titer and days post infection on *Aedes albopictus* ZIKV infection (A), dissemination (B), transmission (C) and transmission efficacy (D) rates.

A) Final logistic model: infection ~ population + temperature + virus titer + day post infection + population:temperature.

|  | **ZIKV infection** | | | **Multiple logistic regression** | | | | |
| --- | --- | --- | --- | --- | --- | --- | --- | --- |
| **Variables** | **Negative (%)** | **Positive (%)** | **Total** | **z-statistic** | **p-value** | **OR** | **Lower 95% CI** | **Upper 95% CI** |
| **Population** |  |  |  | 1.29 | 0.19 | 1.52 | 0.80 | 2.90 |
| MAN_AB | 523 (87.2) | 77 (12.8) | 600 |  |  |  |  |  |
| URC_AB | 523 (87.2) | 77 (12.8) | 600 |  |  |  |  |  |
| **Temperature** |  |  |  | 4.21 | < 0.01 | 3.79 | 2.04 | 7.04 |
| 22ºC | 544 (90.7) | 56 (9.3) | 600 |  |  |  |  |  |
| 28ºC | 502 (83.7) | 98 (16.3) | 600 |  |  |  |  |  |
| **Virus titer** |  |  |  | 11.74 | < 0.01 | 7.61 | 5.42 | 10.67 |
| 10^2^ | 240 (100) | 0 (0) | 240 |  |  |  |  |  |
| 10^3^ | 240 (100) | 0 (0) | 240 |  |  |  |  |  |
| 10^4^ | 240 (100) | 0 (0) | 240 |  |  |  |  |  |
| 10^5^ | 199 (82.9) | 41 (17.1) | 240 |  |  |  |  |  |
| 10^6^ | 127 (52.9) | 113 (47.1) | 240 |  |  |  |  |  |
| **Day post infection** |  |  |  | not included in the final logistic model | | | | |
| 14 dpi | 529 (88.2) | 71 (11.8) | 600 |  |  |  |  |  |
| 21 dpi | 517 (86.2) | 83 (13.8) |  |  |  |  |  |  |
| **Population:temperature** |  |  |  | -1.72 | 0.08 | 0.47 | 0.20 | 1.11 |

B) Final logistic model: dissemination ~ temperature + virus titer.

|  | **ZIKV dissemination** | | | **Multiple logistic regression** | | | | |
| --- | --- | --- | --- | --- | --- | --- | --- | --- |
| **Variables** | **Negative (%)** | **Positive (%)** | **Total** | **z-statistic** | **p-value** | **OR** | **Lower 95% CI** | **Upper 95% CI** |
| **Population** |  |  |  | not included in the final logistic model | | | | |
| MAN_AB | 54 (70.1) | 23 (29.9) | 77 |  |  |  |  |  |
| URC_AB | 56 (72.7) | 21 (27.3) | 77 |  |  |  |  |  |
| **Temperature** |  |  |  | 2.07 | 0.04 | 2.34 | 1.05 | 5.24 |
| 22ºC | 45 (80.4) | 11 (19.6) | 56 |  |  |  |  |  |
| 28ºC | 65 (66.3) | 33 (33.7) | 98 |  |  |  |  |  |
| **Virus titer** |  |  |  | 3.01 | 0.002 | 5.52 | 1.81 | 16.80 |
| 10^2^ | 0 (0) | 0 (0) | 0 |  |  |  |  |  |
| 10^3^ | 0 (0) | 0 (0) | 0 |  |  |  |  |  |
| 10^4^ | 0 (0) | 0 (0) | 0 |  |  |  |  |  |
| 10^5^ | 37 (90.2) | 4 (9.8) | 41 |  |  |  |  |  |
| 10^6^ | 73 (64.6) | 40(35.4) | 113 |  |  |  |  |  |
| **Day post infection** |  |  |  | not included in the final logistic model | | | | |
| 14 dpi | 52 (73.2) | 19 (26.8) | 71 |  |  |  |  |  |
| 21 dpi | 58 (69.9) | 25 (30.1) | 83 |  |  |  |  |  |

C) It was not possible to fit logistic regression models to *Aedes albopictus* transmission since only five specimens were found saliva-positive for ZIKV.

|  | **ZIKV transmission** | | |
| --- | --- | --- | --- |
| **Variables** | **Negative (%)** | **Positive (%)** | **Total** |
| **Population** |  |  |  |
| MAN_AB | 20 (87.0) | 3 (13.0) | 23 |
| URC_AB | 19 (90.5) | 2 (9.5) | 21 |
| **Temperature** |  |  |  |
| 22ºC | 11 (100) | 0 (0) | 11 |
| 28ºC | 28 (84.8) | 5 (15.2) | 33 |
| **Virus titer** |  |  |  |
| 10^2^ | 0 (0) | 0 (0) | 0 |
| 10^3^ | 0 (0) | 0 (0) | 0 |
| 10^4^ | 0 (0) | 0 (0) | 0 |
| 10^5^ | 4 (100) | 0 (0) | 4 |
| 10^6^ | 35 (87.5) | 5 (12.5) | 40 |
| **Day post infection** |  |  |  |
| 14 dpi | 17 (89.5) | 2 (10.5) | 19 |
| 21 dpi | 22 (88.0) | 3 (12.0) | 25 |

D) It was not possible to fit logistic regression models to *Aedes albopictus* transmission efficacy since only five specimens were found saliva-positive for ZIKV.

|  | **ZIKV transmission efficacy** | | |
| --- | --- | --- | --- |
| **Variables** | **Negative (%)** | **Positive (%)** | **Total** |
| **Population** |  |  |  |
| MAN_AB | 597 (99.5) | 3 (0.5) | 600 |
| URC_AB | 598 (99.7) | 2 (0.3) | 600 |
| **Temperature** |  |  |  |
| 22ºC | 600 (100) | 0 (0) | 600 |
| 28ºC | 595 (99.2) | 5 (0.8) | 600 |
| **Virus titer** |  |  |  |
| 10^2^ | 240 (100) | 0 (0) | 240 |
| 10^3^ | 240 (100) | 0 (0) | 240 |
| 10^4^ | 240 (100) | 0 (0) | 240 |
| 10^5^ | 240 (100) | 0 (0) | 240 |
| 10^6^ | 235 (97.9) | 5 (2.1) | 240 |
| **Day post infection** |  |  |  |
| 14 dpi | 598 (99.7) | 2 (0.3) | 600 |
| 21 dpi | 597 (99.5) | 3 (0.5) | 600 |

OR= odds ratio, CI = confidence interval.
